# A Nanostructured Microfluidic Artificial Olfaction for Organic Vapors Recognition

**DOI:** 10.1038/s41598-019-55672-z

**Published:** 2019-12-13

**Authors:** Sajjad Janfaza, Eujin Kim, Allen O’Brien, Homayoun Najjaran, Maryam Nikkhah, Taher Alizadeh, Mina Hoorfar

**Affiliations:** 1 University of British Columbia, School of Engineering, Kelowna, Canada; 2 Department of Nanobiotechnology, Faculty of Biological Sciences, Tarbiat Modares University, Jalal Ale Ahmad Highway, Tehran, 14117 Iran; 3 Department of Analytical Chemistry, Faculty of Chemistry, University College of Science, University of Tehran, Tehran, Iran

**Keywords:** Nanoscience and technology, Nanoscale materials, Engineering, Mechanical engineering, Biomarkers

## Abstract

Selective and sensitive detection of volatile organic compounds (VOCs) is of great importance in applications involving monitoring of hazardous chemicals or non-invasive diagnosis. Here, polymethyl methacrylate nanoparticles with acetone recognition sites are synthesized and integrated into a 3D-printed microfluidic platform to enhance the selectivity of the device. The proposed microfluidic-based olfaction system includes two parylene C-coated microchannels, with or without polymer nanoparticles. The two channels are exposed to 200, 400, 800, 2000, and 4000 ppm of VOCs (methanol, ethanol, acetone, acetonitrile, butanone, and toluene), and sensor responses are compared using a 2D feature extraction method. Compared to current microfluidic-based olfaction systems, responses observed between coated and uncoated channels showed an increased recognition capability among VOCs (especially with respect to acetone), indicating the potential of this approach to increase and fine-tune the selectivity of microfluidic gas sensors.

## Introduction

Volatile organic compound (VOCs) emissions originate from many different sources (e.g., industrial processes, transportation activities, and natural resources) and are prevalent in urbanized areas^1^. Long-term exposure to VOCs has been identified as risk factors leading to diseases such as cancer and respiratory illnesses^2^. Despite associated health risks from VOC exposure, these compounds can be utilized as biomarkers for diagnosis of different diseases (e.g., diabetes^3^, Alzheimer’s^4^, cancers^5^). Thus, gas sensing platforms for VOC detection have attracted widespread attention^6^. Advances in microfabrication and nanotechnology have led to the development of highly efficient and miniaturized gas sensors^7^. For instance, studies have indicated that various nanomaterials (such as carbon nanostructures^8^, and gold nanoparticles^9^) have the potential to increase performance of gas sensors due to their unique properties that are not shown at the bulk scale. Among the aforementioned performance parameters, sensitivity and selectivity are the most important criteria for selection of a particular sensor for detection a target VOC or mixture thereof. For the applications mentioned about (as well as the focus of this paper), selectivity precedes sensitivity. The e-nose platforms tackle the selectivity requirement by adding an array for sensor^10^. Along with these benefits, however, the necessity for regular calibration of every individual sensor (due to “sensor drift”) is a challenge. Recently, a new generation of gas detectors was developed by integrating a microfluidic channel with a single sensor. These detectors have increased selectivity over their single sensor counterparts; this is primarily due to the effect of diffusion and adsorption/desorption (highly influenced by the coating materials and dimensions of the microfluidic channel) of the analyte molecules along the microfluidic channel^11–13^. Therefore, the combination of the diffusion and adsorption/desorption rates of different analytes with different concentrations leads to a unique response (i.e., “smell print”) from the embedded sensor^14^. The theory and mechanism of the detector have been extensively discussed previously^13^. A recent study^15^ has shown that coating of the microchannel needs to be adjusted depending on the polarity of the target analyte (or mixture). In addition to the coating (or the combination of the layers coated) the surface roughness can affect adsorption and desorption phenomena and hence the performance (mainly selectivity) of the sensors. There are several ways to change the coating and roughness of the channel (such as adding nanostructures^16^). One potential approach, which changes the surface material of the microchannel and interaction with the analyte, is the integration of molecularly imprinted polymer nanoparticles (MIP NPs) into the channel.

MIP is a synthetic polymer possessing selective molecular recognition properties (due to its binding sites) with size, shape, and functionalities complementary to the target molecules. MIPs, often called artificial antibodies or receptors, are alternatives to antibodies in a variety of applications^17^. The advantages of MIPs over natural antibodies include easy and low-cost preparation, longer life cycles (up to several years), highly tailorable recognition properties, solvents and thermal stability, robustness and resistance to a wide range of pH, and ease of regeneration and sterilization^18,19^. MIPs have been recently integrated with microfluidic chip for a wide range of applications^20–22^. Studies have shown that the functionality of MIPs can be further improved in nanoscale dimensions^23^ due to an increase in chemical reactivity, their higher specific surface area, binding sites to target molecules, uniform spherical geometry and increased binding capacity and kinetics in comparison to bulk MIPs.

Here, for the first time, MIP NPs were synthesized with acetone recognition sites due to its importance as a biomarker for the non-invasive diagnosis of several diseases like diabetes^24,25^. The synthesized MIP NPs were integrated into a 3D-printed dual-channel platform (one channel without and the other with MIP NPs) to develop a highly selective detection device against various VOCs (alcohols, ketones, nitrile, and aromatic compounds). A simple feature extraction method was used to distinguish signals arising from different VOCs. The differences between the feature extraction of the two channels showed that in general this platform was specific to acetone, proving the potential application of the MIP NPs in artificial olfaction platforms.

## Results and Discussion

### Characterization of MIP NPs

**Figure 1:**
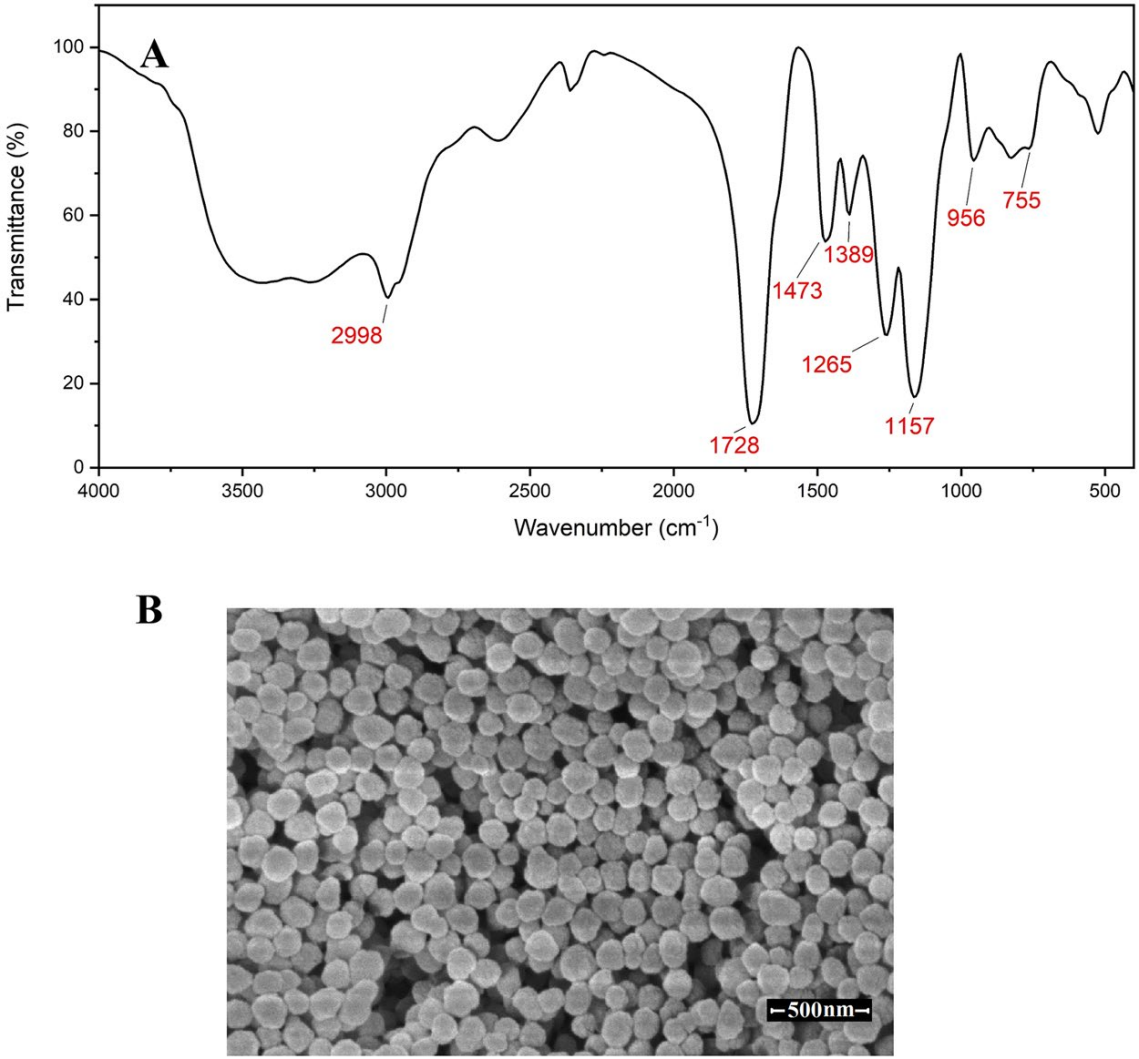
The MIP nanoparticles characterization; (**A**) FTIR spectra and (**B**) SEM photographs of synthesised MIP nanoparticles demonstrate that uniform MIP nanoparticles have been successfully synthesized.

FTIR analysis of MIP NPs was recorded in the range of 4000–500 cm^−1^ (Fig. 1(A)). The strong spike at 1157 cm^−1^ and band at 1728 cm^−1^ are most likely due to C-O-C and C = O stretching vibration of the cross-linker (EGDMA), respectively. The stretching vibrations of OH of carboxyl groups (3000–3500 cm^−1^) and carbonyl groups (at about 1730 cm^−1^) were the characteristic absorption of COOH of MAA. The bands at 755, 1389, 1473, and 2998 cm^−1^ are attributed to the C-H vibrations. In addition, no peak was observed at around 2240 cm^−1^ (radical AIBN). The above-mentioned results demonstrate that MIP NPs were successfully synthesized.

The morphology of MIP NPs was investigated by SEM (Fig. 1(B)). The average size of the spherical MIP NPs was approximately 123 ± 17 nm. Spherical MIP NPs with uniform size have increased the interaction with a template molecule; thereby it is expected to enhance the recognition capability of the microfluidic gas sensor when MIP NPs are coated along the microfluidic channel. Moreover, the small size of MIP NPs increases the accessibility of VOCs to the binding cavities due to a higher specific surface area, and also allows for rapid removal of VOCs from the cavities.

### Analytical characterization of microfluidic gas detectors

The response of the detector depends on the type and concentration of the target analytes diffusing through the microchannel and reacting with the sensing layer of the MOS sensor. Previous studies have demonstrated^13^ that channel dimensions and surface coatings influence analyte diffusion and adsorption/desorption. The adsorption and desorption rate of VOCs to or from the channel walls are dependent on the both gas type and the channel surface material. Based on the above-stated reasons, it is expected that the surface modification of the microchannel with MIP NPs leads to different responses. In fact, we believe that the comparison between the responses from the MIP-coated and bare channel can significantly improve selectivity of the microfluidic gas detector due to the difference between the surface interaction of the analyte and two channels (the MIP-coated channel adds the specificity of the binding sites towards the target analyte).

**Figure 2:**
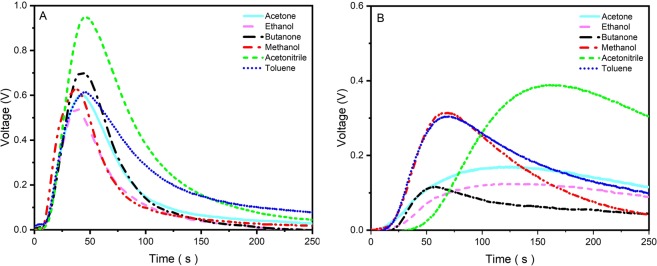
The response of (**A**) bare (control detector without MIP NPs treatment), and (**B**) MIP-coated detector exposed to 800 ppm of different VOCs.

The responses of the dual-channel detector towards 800 ppm of various VOCs (including acetone, ethanol, methanol, acetonitrile, butanone, and toluene) are shown in Fig. 2. The results obtained from the control detector (see Fig. 2(A)) for different analytes show that the shape of the majority of the response curves are similar (with different maximum values around the same time). However, the responses (both in terms of the magnitude and shape) of the detector including the MIP NPs are significantly dissimilar between different analytes (Fig. 2(B)). This observed difference, which is due to the interaction of the analytes with the MIP NPs, provides a higher degree of selectivity for the detector. In essence, the interaction between the MIP NPs and VOCs can be described by $${\rm{VOC}}+{\rm{MIP}}\begin{array}{c}{K}_{1}\\ \,\rightleftarrows \,\\ {K}_{2}\end{array}{\rm{VOC}}-{\rm{MIP}}$$, where *K*_1_ and *K*_2_ are adsorption and desorption rate constants, respectively. The higher affinity of VOCs to the MIP NPs causes the larger values of *K*_1_, leading to a higher adsorption rate and a decrease in the transport rate of VOCs through the channel.

The simple structure of the MIP NPs’s binding sites offers two advantages for the proposed artificial olfaction system: (1) No channel heating for promoting desorption of the target analyte from the MIP NPs cavities (during the recovery phase) is required since the interaction between VOCs and the MIP NPs’s binding site is physical adsorption which allows the complete recovery of the MIP-coated channel in clean air. Chemical adsorption of analytes into the channel wall prevents the complete recovery of the channel. We did not observe any partial recovery (see Figure S1 showing complete recovery at the end of each cycle recovery). (2) Molecules structurally similar to the template molecule can interact with the binding sites, making the detector selective to a wide range of analytes based on their affinity to MIP NPs. Among different analytes tested with the MIP NPs treated detector, the smell print curves of acetone, ethanol, and butanone have lower magnitude at the peak compared to other VOCs which have less similar structures to the template molecules (acetone). There are three possible factors that can affect the smell prints observed in Fig. 2(B): (i) the MOS reaction to analytes, (ii) trapping of the analytes in the MIP NPs’ cavities, and (iii) the level of interaction (strong versus weak) of analytes with the MIP NPs. For ethanol, the shape of the smell print curve during both exposure and recovery periods is similar to that of acetone, which indicates a similar level of interaction with the binding sites for these two analytes. The template molecule (acetone) used in this study has a low number of functional groups (only one carbonyl group), and hence the other chemically similar molecules, like ethanol, can also be trapped in the cavities and hence interact with the binding sites. For butanone, the low magnitude of the voltage indicates the interaction with the binding sites (as butanone has a similar structure to the template molecules); however, the recovery is much faster as compared to acetone and ethanol responses. This could be due to the weak level of the interaction between butanone and the binding sites. The comparison of two channels’ responses to acetonitrile demonstrates that the developed gas detector exhibits highest responses to acetonitrile among other exposed VOCs. In addition, the long retention time for the MIP-coated channel while exposed to acetonitrile indicates the interaction between MIP NPs and acetonitrile. This potential interaction can be attributed to the presence of the functional groups (like hydroxyl groups) on the surface of MIP NPs. Therefore, the response of the detector to acetonitrile can mostly be influenced by either the MOS sensor and/or the interaction of acetonitrile with the microchannel wall. As for methanol and toluene, it seems that they are not trapped in the cavities and hence not interacting with the binding sites (they are either much smaller or larger than the cavities). As compared to methanol, the smell print of toluene has a slower recovery time which is attributed to the large size of toluene.

In addition to the type of analyte, the effect of concentration on the detectors’ response has been studied. Figure S2 shows the response of the dual-channel detector when exposed to 200, 400, 800, 2000, 4000 ppm concentrations of several VOCs. The responses of both detectors increase with increasing concentrations of the target VOC (as shown before in^13^). Although the response of the two detectors to the same target analyte was significantly different, with the increase in the concentration the magnitude of signals increases; however, the time of the maximum response still remains the same. It was also observed that for the low concentration of acetone, butanone and ethanol, the responses from the nonoMIP-coated detector are not distinguishable. This again confirms the interaction between these analytes and the MIP NPs.

**Figure 3:**
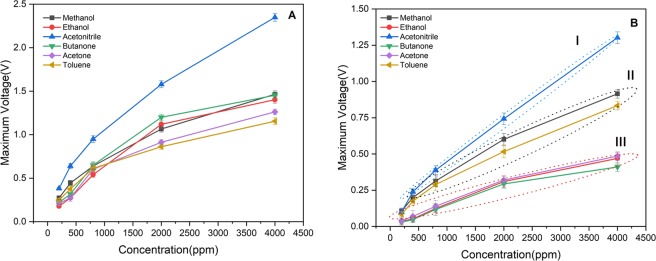
The calibration curve of (**A**) non-coated and (**B**) MIP-coated detectors to different concentrations of methanol, ethanol, acetonitrile, butanone, acetone, and toluene.

To compare the effect of the analyte concentration on the responses of the two detectors, the values at the peak were plotted as a function of the analyte concentrations (Fig. 3). The results show no patterns in the responses of the control detector to different VOCs; whereas for the MIP-coated detector, there are clearly three distinct clusters of curves including acetonitrile (cluster I), methanol and toluene (cluster II), and acetone, ethanol, and butanone (cluster III), which confirms the discussion provided before.

### Feature extraction and response analysis of dual-channel gas detector

**Figure 4:**
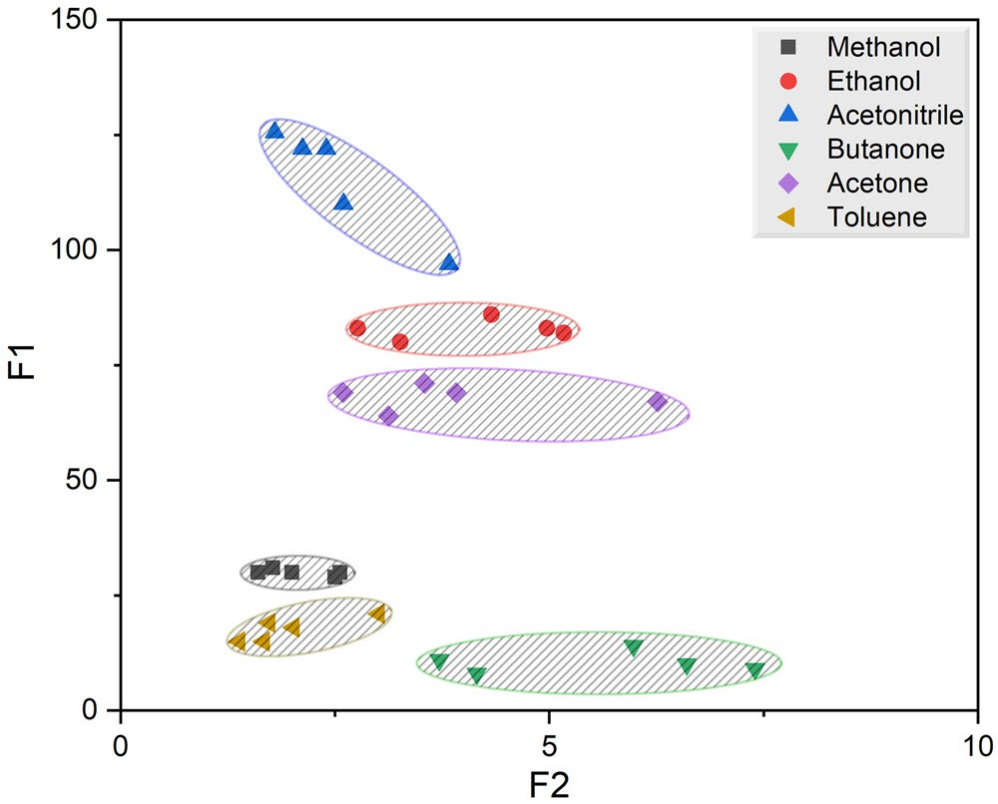
The 2D feature space presentation for all the responses presented in Figure S2. F1 and F2 are the difference between the time of the maximum responses of the bare and MIP-coated channel detectors and the ratio of the maximum responses of the two detectors, respectively.

After obtaining the response curves, a feature extraction method was used for pattern recognition in a 2D space (Fig. 4). Each point presents features for a certain concentration of a certain analyte. The separation of different analytes has been achieved in this 2D-feature plots that clearly demonstrates the selectivity of the proposed dual-channel platform.

Sensors’ selectivity is measured by the magnitude of the Euclidean distance between the analytes. The larger the Euclidean distance between two groups, the greater the difference between them and hence higher selectivity. The Euclidean distances between acetone, the template molecule, and the average distribution of target analytes are butanone > toluene > acetonitrile > methanol > ethanol (see Table 1). Ethanol is the closest (which means less selectivity between acetone and ethanol) due to similarity in its structure to the template molecule (as explained in the previous section). Based on this order, the butanone distribution average has the largest distance from acetone.

**Table 1:** The Euclidean distances between the average feature vectors in the 2D feature space.

	Methanol	Ethanol	Acetonitrile	Butanone	Acetone	Toluene
Methanol	0	52.8	85.3	19.9	38.0	12.4
Ethanol	52.8	0	32.5	72.4	14.8	65.2
Acetonitrile	85.3	32.5	0	104.9	47.3	97.7
Butanone	19.9	72.4	104.9	0	57.6	8
Acetone	38	14.8	47.3	57.6	0	50.4
Toluene	12.4	65.2	97.7	8	50.4	0

To reduce the effect of each analyte’s distribution on the sensor’s selectivity measurement, Mahalanobis distances were calculated from the mean of a certain analyte to the distribution of another. The Mahalanobis distance increases only due to the true distance between the distributions, and unlike the Euclidian distance, is not affected by the lack of precision (scatter) of features of the analytes. Thus, the greater the distance between two distributions or the higher the precision (compactness) of the features of the analytes the greater the Mahalanobis distance. Table 2 lists the Mahalanobis distances between the mean of an analyte to the distribution of another analyte. For instance, the Mahalanobis distances from the mean distribution of acetone to different distributions is ordered as methanol > toluene > butanone > acetonitrile > ethanol. Again, ethanol, is the closest to acetone. However, methanol has the largest Mahalanobis distance to acetone. It is noted that this is due to the fact that the distribution of methanol is least scattered in Fig. 4.

**Table 2:** The Mahalanobis distances between the average (mean) feature vector of each analyte and the distribution of another analyte group (shown as a cluster in Fig. 4) in the 2D feature space.

	Methanol (Distribution)	Ethanol (Distribution)	Acetonitrile (Distribution)	Butanone (Distribution)	Acetone (Distribution)	Toluene (Distribution)
Methanol (Mean)	0	607.9	808.7	79.6	214.3	73.4
Ethanol (Mean)	9371.1	0	13	996	31	1718
Acetonitrile (Mean)	22920	250.9	0	2096.5	320.8	4692
Butanone (Mean)	879.9	1203.6	366	0	475.3	235.2
Acetone (Mean)	4946.1	48.6	76.1	631.9	0	985.8
Toluene (Mean)	488.5	930.3	1077.7	15.8	374.6	0

## Conclusions

Here we have introduced the first working prototype of a dual-channel olfaction platform coated with MIP NPs to enhance the selectivity of microchannels in detecting specific analytes. By successfully synthesizing uniform MIP NPs (with a size of around 120 nm) for acetone, we have been able to finetune the selective process of artificial olfaction sensors.

The effectiveness of the MIP NPs was studied by comparing the response curves obtained from two channels. The results showed that MIP NPs enhance selectivity due the analyte interaction with the surface and binding site of these nanoparticles. Other molecules with a similar size and shape (e.g., ethanol) to those of acetone can fit in MIP NPs cavities and interact with them, resulting in a low magnitude and similar-shape response curve as that of acetone. However, this simple structure of the MIP NP’s binding sites eases the recovery process of the sites and makes it applicable to a wide range of analytes based on their affinity to MIP NPs. A simple feature extraction method was used based on the significant difference between the responses of the two channels (coated vs uncoated). Both Euclidean and Mahalanobis distances were used to analyze the separation capability and hence selectivity of the proposed platform. The results showed great selectivity of the proposed platform between the template molecule (acetone) and the other VOCs. However, since the template molecule has a low number of functional groups, the other chemically similar molecules like ethanol can interact with imprinted sites in MIP NPs and that may lead to some cross-sensitivity. This issue was addressed by using a 2D feature extraction method.

In conclusion, we achieved a better recognition performance between analytes compared to the control channel by tailoring the MIP NPs. The proposed artificial olfaction system was able to effectively distinguish acetone from the molecules with even a very similar chemical structure. This has important implications in the development of MIP-based olfaction systems that are able to discriminate different volatile organic compounds. Finetuning has a great impact on these sensors and offers directions for future research. This strategy will help filter out off-targets which are considered as “noise”.

## Methods

### Materials

Methacrylic acid (MAA, 99%), ethylene glycol dimethacrylate (EGDMA, 98%), 2,20-azobisisobutyronitrile (AIBN, 98%), acetone (≥ 99.5%), acetonitrile (99.8%), 2-Butanone (99.0%), methanol (≥ 99.9%), ethanol (99.9%), and toluene (99.8%) were purchased from Sigma-Aldrich. Parylene C (poly (p-xylylene) polymer, CAS No: 28804-46-8) was obtained from Specialty Coating Systems (Indianapolis, IN).

### Synthesis of molecularly imprinted polymer nanoparticles

The MIP NPs with acetone recognition sites were prepared for the first time using the simple precipitation procedure^26^. Briefly, methacrylic acid (1 mmol), acetone (1 mmol) and 40 mL of dry acetonitrile were placed into a 100 mL round-bottomed flask, and the mixture was incubated for 20 min. Subsequently, EDMA (3 mmol) and AIBN (70 mg) were added. The mixture was purged with nitrogen for 15 min. The mixture of chemicals was sealed in a glass bottle and thermally polymerised in oil bath at 60–65 °C for 16 h under stirring conditions (at 300 rpm). After polymerization, the solution was centrifuged and the final polymer was ground into a powder. To remove the template, the MIP powder was washed with acetonitrile, followed by heating the powder at 80 °C for 48 h. The morphology and chemical structure of MIP NPs were investigated by field emission scanning electron microscope (FE-SEM, FEI-Nova NanoSEM 450) and Fourier-transform infrared spectroscopy (FTIR, Shimadzu FTIR Prestige 21).

### Fabrication of microfluidic-based gas sensor

The microfluidic-based gas platform consists of two microfluidic channels and gas sensors (embedded at the channel terminus). The microchannels were fabricated from VeroClear RGD810 using a 3D-printer (Connex 500). Each microchannel has equal dimensions of 500 µm in height, 3 mm in width, and 3 cm in length. The inner surfaces of the microchannels are coated with Parylene C using a chemical vapor deposition (CVD) coating machine (SCS PDS 2010 Labcoater). To investigate the effect of treating the channel with MIP NPs on the selectivity of the sensors, one channel was coated with MIP NPs (referred to as the MIP channel) and another one was left uncoated (referred to as the control channel). For this purpose, a certain amount of synthesized MIP NPs dispersed in acetonitrile was drop-casted on the surface of the MIP channel and dried for a day in a clean air chamber. VOCs flow into the channel inlet, diffuse along the microfluidic channel, and reach gas detectors placed at the end of the microchannels, when exposed to the device. Figures S3 and S4, supporting information, shows the schematic illustration and photo of the MIP-based microfluidic sensor respectively.

### Measurement and sampling system

A commercially available metal oxide semiconductor (MOS)-based chemiresistive sensors (TGS2600, Figaro, Japan) was used. To obtain the ideal operating temperature (300 °C), 5 V of DC was applied to the sensor’s microheater. The electrical properties of the sensor were measured by using the Science Workshop 750 interface data acquisition system with Datastudio software. The output voltage of the gas detector was measured using a conventional circuit (Figure S5(A), supporting information) at a circuit voltage of 5 V. The electrical resistance of the sensor (*R*_*s*_) can be defined as ${R}_{s}=\frac{{V}_{in}-{V}_{out}}{{V}_{out}}{R}_{l}$, where *V*_*out*_ and *V*_*in*_ are the output and applied voltages, respectively, and *R*_*l*_ is the resistances of the external resistor (Figure S5, supporting information).

The selectivity of the detectors towards several VOCs was investigated using a static system that allowed the generation of different vapor concentrations. The target analyte (in a liquid phase) were first injected into a fixed volume chamber (10 L) using a precise Pipet-Lite XLS microsampler (Figure S6, supporting information) and was let to evaporate. After ensuring uniform distribution of analyte vapor in the test chamber (using a fan), the detector was exposed to VOCs inside the test chamber for 30 s and then it was recovered in clean air for 220 s (Figure S5(B), supporting information). The electrical resistance of the sensor during exposure to the target gas and recovery in clean air was obtained as a function of time.

The same experimental procedure carried out to investigate the response of the dual-channel detector to VOCs (including of acetone, ethanol, methanol, acetonitrile, butanone, and toluene) at the same (800 ppm) and different concentrations (200, 400, 800, 2000, 4000 ppm). The testing chamber was purged with clean air before each experiment. All experiments were performed at 25 °C and relative humidity of 40%.

### Feature extraction and response analysis

Feature extraction methods^27^ have been used to analyze sensors’ responses and relate them to the type and concentration of target analytes. Examples of the features reported in literature^13^ include: the magnitude and time of the maximum response, the difference between maximum and minimum responses, the area underneath the response curve, the slope of the response curve. Here, the significant differences between smell prints obtained from the bare (control) and MIP-coated channels are used as features. More specifically, the difference between the peak time of the bare channel and that of MIP-coated channel detectors (presented as *F*1), and the ratio of the peak responses of the two detectors (presented as *F*2) are used as features. The Euclidean^28^ and Mahalanobis^29^ distances (Supplementary Information) are used as a way to quantitatively analyze the separation capability and the selectivity of the proposed platform. In essence, the former shows the distance between the mean (average) of the distribution of each analyte and that of another analyte in the 2D feature space. The latter shows the distance between the mean of an analyte distribution and the distribution of another analyte normalized by the variance to account for the scattered data in the distribution.

## Supplementary information

Supplementary Information
