# Supplementary material for: A Nanostructured Microfluidic Artificial Olfaction for Organic Vapors Recognition
Source: Sci Rep. 2019 Dec 13;9:19051. doi: 10.1038/s41598-019-55672-z (PMC6911096; doi:10.1038/s41598-019-55672-z)
Supplement: Supplementary file 1 — Supplementary Information [file 41598_2019_55672_MOESM1_ESM.docx]

Supporting Information

A Nanostructured Microfluidic Artificial Olfaction for Organic Vapors Recognition

Sajjad Janfaza, Eujin Kim, Allen O'Brien, Homayoun Najjaran, Maryam Nikkhah,* Taher Alizadeh, Mina Hoorfar*


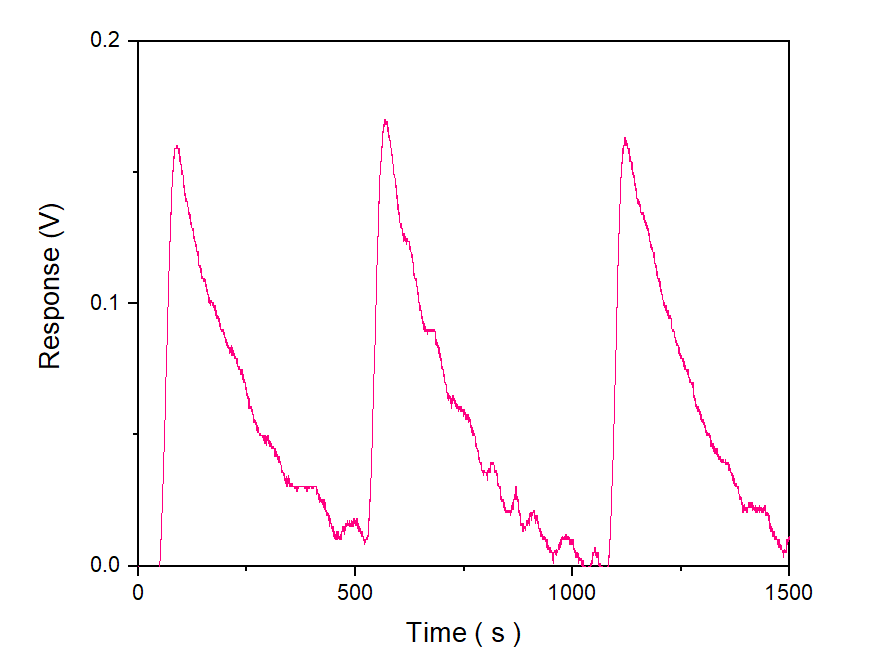


**Figure S1.** The time response characteristics for three consecutive cycles of the MIP-coated microfluidic channel to acetone. The results shows complete recovery after each cycle.

**
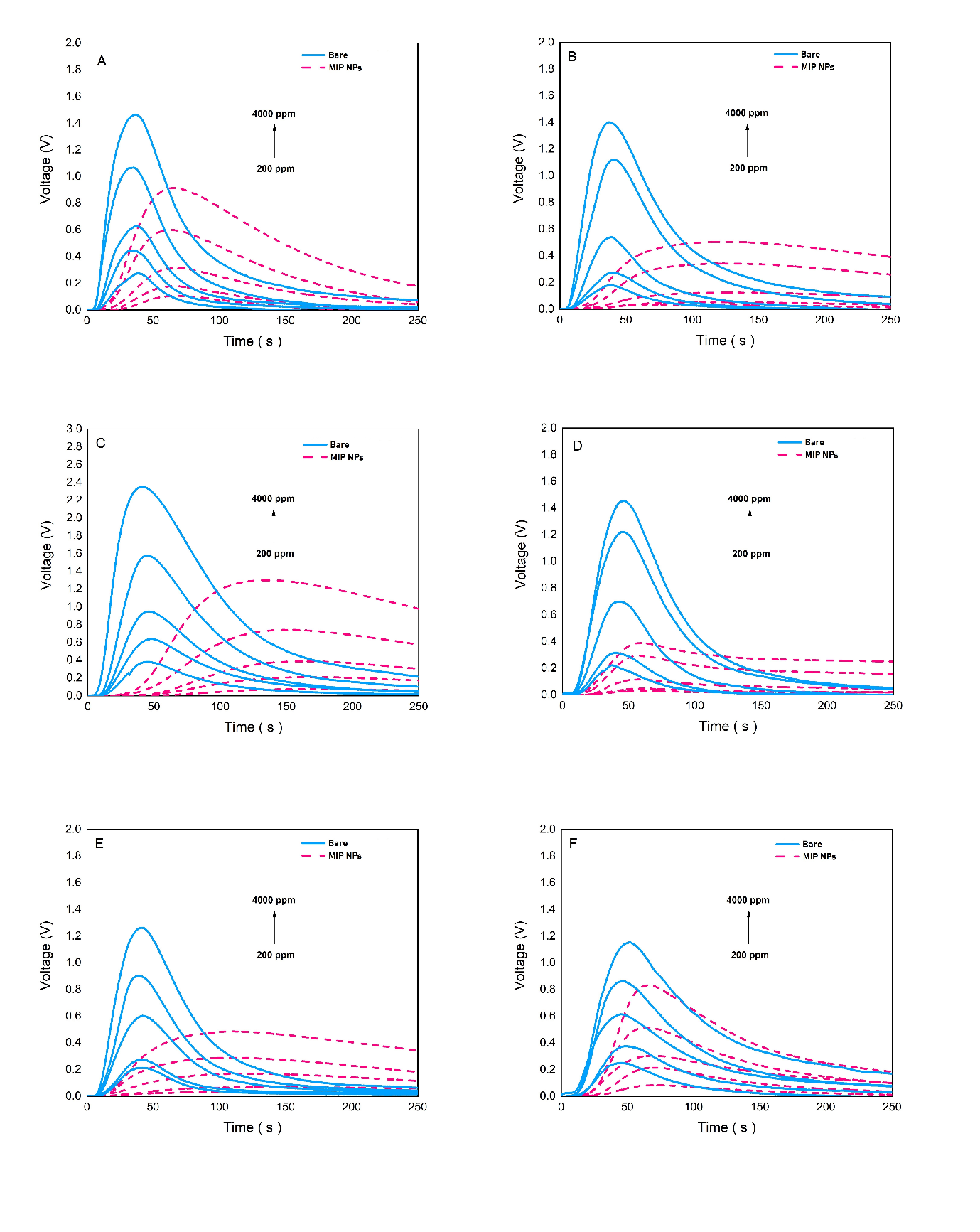
**

**Fig. S2.** Transient responses of the dual-channel detector to five different concentrations (200 ppm–4000 ppm) of 6 different analytes including: (A) methanol, (B) ethanol, (C) acetonitrile, (D) butanone, (E) acetone, and (F) toluene.


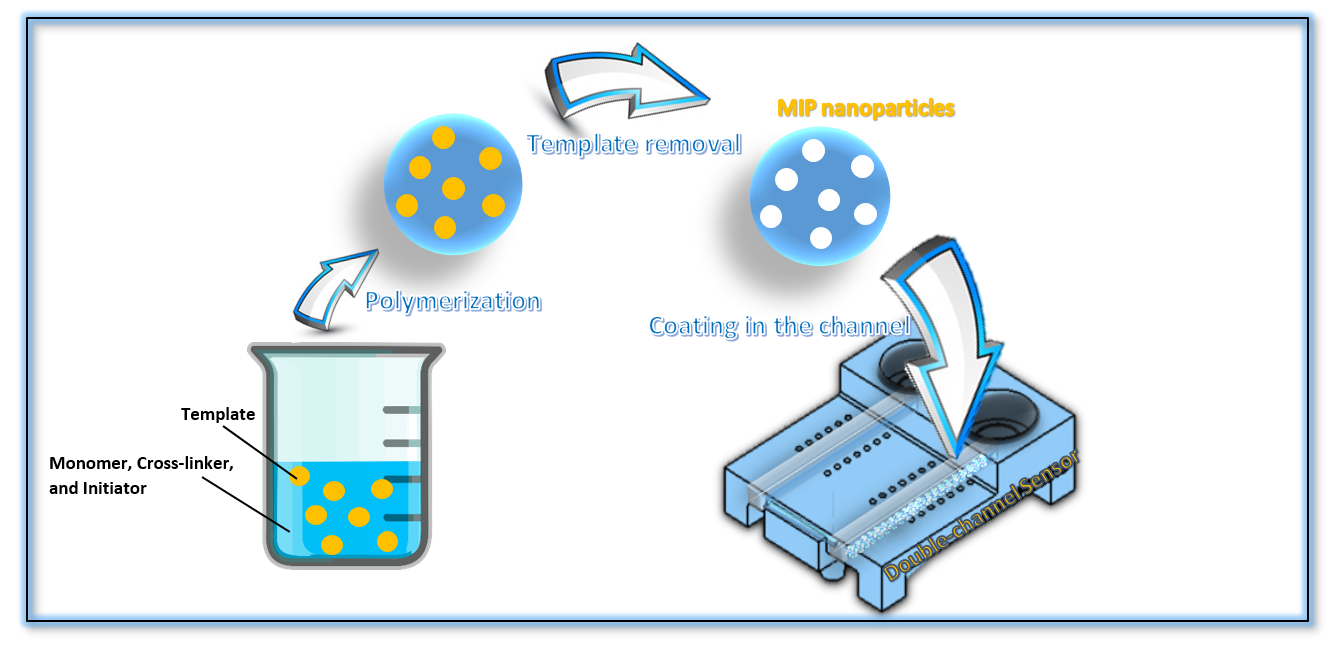


**Figure S3:** The scheme of the polymerization approach and development of the nanoMIP-based microfluidic gas detector. For nanoMIPs synthetization, monomer units are assembled around a template molecule and subsequently polymerized using a cross-linker. Then, the template is removed to create cavities within the MIPs.


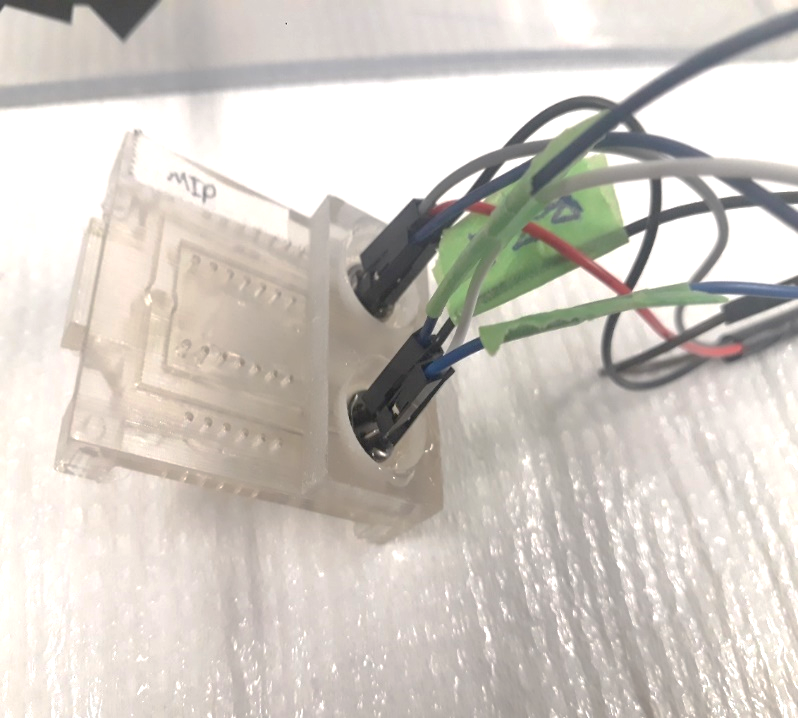


**Figure S4:** The photograph of the 3D-printed microfluidic sensor.


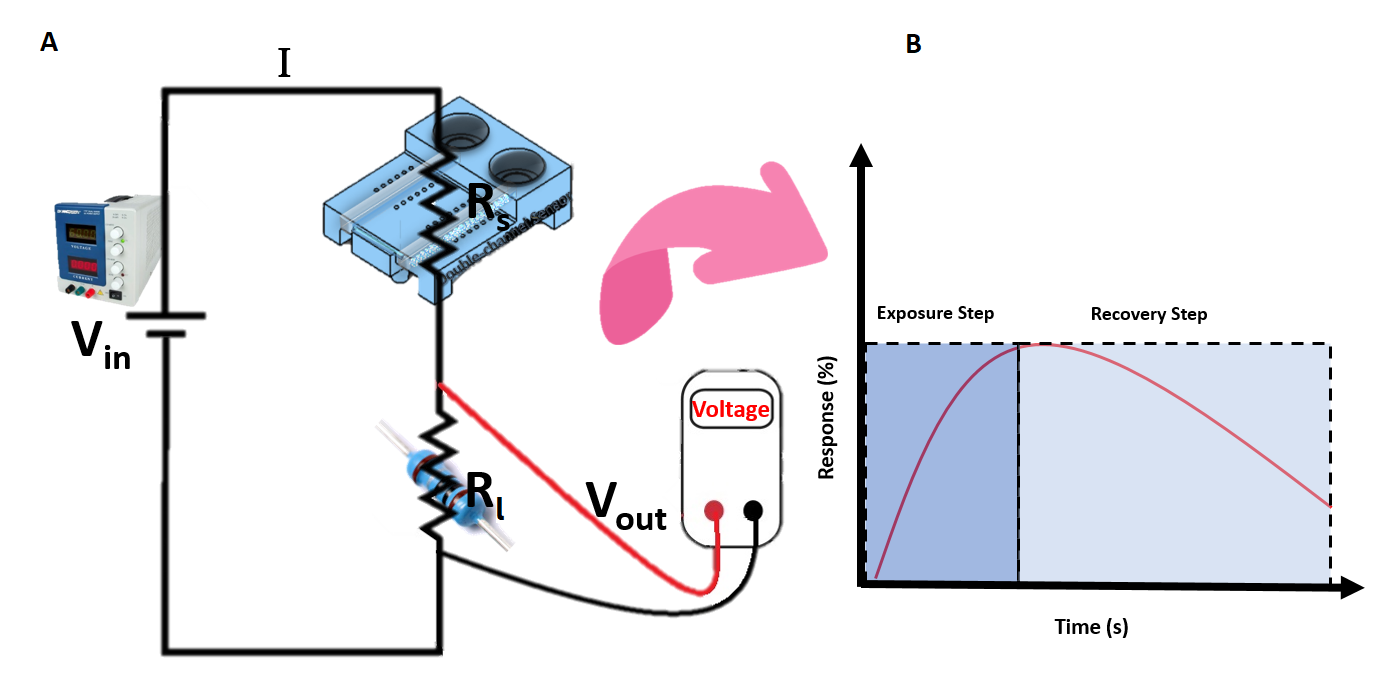


**Figure S5:** (A) The schematic diagram of the MOS gas sensor electric circuit used for measuring the voltage as a function of time during the exposure and recovery periods. (B) A typical response of the microfluidic-based gas detector containing the exposure and recovery phases.


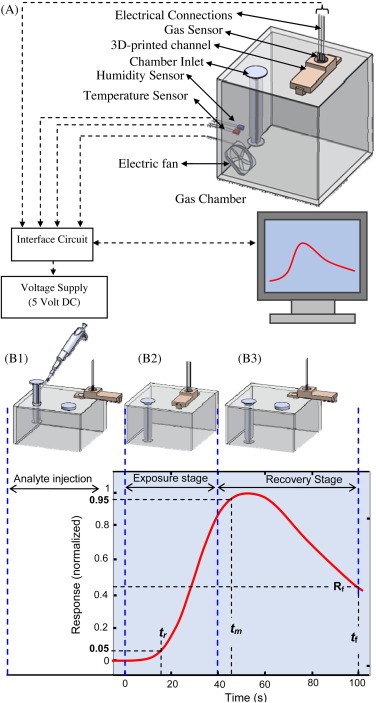


**Figure S6.** (A) The schematic of the experimental setup. The sensor is mounted on a chamber. (B) Three different phases of an experiment and a typical normalized [transient response](https://www.sciencedirect.com/topics/engineering/transient-analysis) of the sensor to a concentration of a [gas](https://www.sciencedirect.com/topics/chemistry/gas) are shown; B1: analyte injection stage, B2: exposure stage, and B3: recovery stage. Reproduced from [13] with permission from Elsevier, copyright 2017”

**Euclidean distances**

To compare quantitatively the selectivity of the device toward different analytes, the Euclidean distances of the average feature vectors were calculated for each pair of the examined analytes in the feature space using following Equation:

$D=\sqrt{{({Avg F1}_{i}-{Avg F1}_{J})}^{2}+{({Avg F2}_{i}-{Avg F2}_{J})}^{2}}$ (1)

where $F1$ and $F2$ are the distance between time of the maximum responses of two detectors and the ratio of the maximum response of the bare channel to that of the nanoMIP-coated channels, respectively. $i,j=a,b,c,d,e$, or $f$ refer to methanol, ethanol, propanol, acetone, acetonitrile, and butanone, respectively.

**Mahalanobis distances**

Mahalanobis distances were also calculated for each pair of the examined analytes. Equation (2) shows the Mahalanobis distance between two vectors, x and y:

$d_{M}(x,y)=\sqrt{\left( x-y \right)^{T}S^{-1}(x-y)}$ (2)

where $S$ is the covariance matrix.
